# Peroxisome Proliferator-Activated Receptor-*γ* Is Critical to Cardiac Fibrosis

**DOI:** 10.1155/2016/2198645

**Published:** 2016-05-12

**Authors:** Huang-Jun Liu, Hai-Han Liao, Zheng Yang, Qi-Zhu Tang

**Affiliations:** ^1^Department of Cardiology, Renmin Hospital of Wuhan University, Wuhan 430060, China; ^2^Cardiovascular Research Institute of Wuhan University, Wuhan 430060, China; ^3^Hubei Key Laboratory of Cardiology, Wuhan 430060, China

## Abstract

Peroxisome proliferator-activated receptor-*γ* (PPAR*γ*) is a ligand-activated transcription factor belonging to the nuclear receptor superfamily, which plays a central role in regulating lipid and glucose metabolism. However, accumulating evidence demonstrates that PPAR*γ* agonists have potential to reduce inflammation, influence the balance of immune cells, suppress oxidative stress, and improve endothelial function, which are all involved in the cellular and molecular mechanisms of cardiac fibrosis. Thus, in this review we discuss the role of PPAR*γ* in various cardiovascular conditions associated with cardiac fibrosis, including diabetes mellitus, hypertension, myocardial infarction, heart failure, ischemia/reperfusion injury, atrial fibrillation, and several other cardiovascular disease (CVD) conditions, and summarize the developmental status of PPAR*γ* agonists for the clinical management of CVD.

## 1. Introduction

Cardiac fibrosis is an inevitable process of varieties of cardiovascular diseases (CVDs) and is characterized by abnormal accumulation of extracellular matrix (ECM) in the myocardial interstitium. The ECM, composed of collagens, elastic fibers, glycosaminoglycan, and glycoproteins [1], are derived mainly from fibroblasts. Under physiological conditions, ECM is necessary to maintain the normal structure and function of the heart, the formation and degradation of ECM retain in dynamic balance, while in pathological conditions, because of excessive activation of renin-angiotensin-aldosterone system (RAAS), maladjustment of matrix metalloproteinases (MMP), and excessive secretion of some regulation cytokines such as transforming growth factor beta (TGF*β*), the dynamic balance would be broken which resulted in ECM deposition and eventually cardiac fibrosis [2]. This pathological process is the beginning of cardiac remodeling and directly leads to arrhythmia [3], impaired cardiac function [4, 5] heart failure (HF), and even sudden cardiac death [6].

Although there are no effective strategies for treatment of cardiac fibrosis right now, it is firmly convinced that inhibition or reversion of myocardial fibrosis will be a promising way for prevention and treatment of HF in the nearby future [7]. Currently, the strategies for treatments of cardiac fibrosis mainly target RAAS system and inflammatory response; however, more and more other molecular mechanisms have been recognized to involve the regulation of cardiac fibrosis [8].

Interestingly, peroxisome proliferator-activated receptor-*γ* (PPAR*γ*) has been identified to have the function of antimyocardial fibrosis [9–11]. According to published investigations, PPAR*γ* has a wide spectrum of functions in regulating metabolism, attenuating inflammation, maintaining the balance of immune cells, inhibiting apoptosis and oxidative stress, and improving endothelial function [12]. All of these biological functions will be benefit for preventing the cardiac function from deterioration. However, the underling mechanisms of PPAR*γ* in the regulation of cardiac fibrosis are not fully illustrated yet. This review will mainly summarize the reports about PPAR*γ* and its agonist in the regulation of cardiac fibrosis.

## 2. Structure and Function of PPAR*γ*

**Figure 1:**
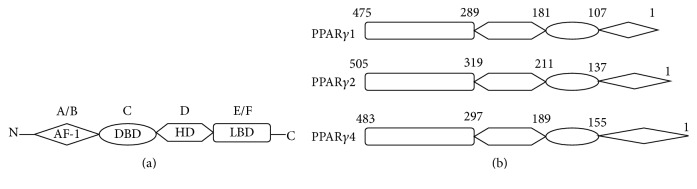
Schematic structure of peroxisome proliferator-activated receptor-*γ* and its protein isoforms. A/B, C, D, and E/F indicate the N-terminal A/B domain containing a ligand-independent AF-1, the DNA-binding domain, the hinge region, and the C-terminal LBD containing AF-2, respectively. AF-1 is responsible for phosphorylation, while AF-2 promotes the recruitment of coactivators for gene transcription. PPAR: peroxisome proliferator-activated receptor; AF: activation function; DBD: DNA-binding domain; HD: hinge domain; LBD: ligand-binding domain. Figure adapted from [13].

PPARs, belonging to the nuclear hormone receptor superfamily and consisting of three isoforms, PPAR*α*, PPAR*β*/*δ*, and PPAR*γ*, are ligand-inducible transcription factors. They are encoded by three separate genes and are distributed in different organs and tissues [14]. Because of the different expression and distribution profile, each of them presents unique biological function [14–16]. Activated by their specific ligands, PPARs can transfer into nucleus and form heterodimers with the retinoid X receptor. The heterodimeric complexes then band to the promoter region of target genes carrying peroxisome proliferator response elements (PPREs) and regulate transcription of target genes [17, 18]. Being similar to other nuclear receptors, PPAR isoforms possess five or six structural regions within four functional domains [14, 17, 19]. Activation function-1 motif (AF-1) locates at the N-terminal and is the target of phosphorylation kinase. The DNA-binding domain (DBD) consists of two highly conserved zinc finger motifs and is responsible for binding to PPRE. The hinge domain (BD) serves as a docking site for cofactors. The ligand bind domain (LBD) located at the C-terminal (E/F domain) is in charge of ligand specificity and activation of PPARs that bind to the PPRE, which increases target gene expression (Figure 1) [14, 17, 19].

The PPAR*γ* gene is located on human chromosome 3p25 [20]. Seven transcripts have been identified, termed PPAR*γ*1, PPAR*γ*2, PPAR*γ*3, PPAR*γ*4, PPAR*γ*5, PPAR*γ*6, and PPAR*γ*7 [17]. The PPAR*γ*1, PPAR*γ*3, PPAR*γ*5, and PPAR*γ*7 mRNA transcripts translate PPAR*γ*1 protein and PPAR*γ*2 mRNA yields PPAR*γ*2 protein, while PPAR*γ*4 and *γ*6 mRNA transcripts translate PPAR*γ*4 protein [21–23]. Because of different transcript, translation, and tissue distribution, each protein has different biological functions in a variety of organs and cells (Table 1) [13]. So it is not a surprise that PPAR*γ* plays important roles in CVDs including hypertension [17, 24, 25], atherosclerosis [26], HF [27], diabetic cardiomyopathy [11, 28], angiogenesis [29], valvular calcification [30], aortic aneurysm [31], restenosis following cardiovascular interventions [32], and ischemia/reperfusion (I/R) injury [33, 34].

## 3. PPAR*γ* and Cardiac Fibrosis

The primary of activation of PPAR*γ* is to lower serum glucose and improve the insulin sensitivity. In the clinical practice, the specific ligands of PPAR*γ* have been accepted for treatment of diabetes mellitus. However, more and more researches had indicated that activation of PPAR*γ* presents pleiotropic biological effects involving regulation of inflammation and energy metabolism. Because of its pleiotropic effects, PPAR*γ* has been recognized as a target for the treatment of cardiac fibrosis. The characteristics of PPAR*γ* regulate myocardial fibrosis in different CVDs as described below.

### 3.1. Diabetic Cardiomyopathy

The diabetic cardiomyopathy is accompanied by myocardial hypertrophy, dilated ventricular chamber, and fibrosis [49]. The specific PPAR*γ* ligands, thiazolidinediones (TZDs), are used in clinical practice to improve insulin sensitivity in type 2 diabetes mellitus (T2DM). As shown in Table 2, evidences have demonstrated that TZDs could decrease myocardial fibrosis and improve cardiac dysfunction. In the animal experiment, Ihm and his colleagues found that the PPAR*γ* ligand, rosiglitazone, significantly decreased myocardial fibrosis in the Otsuka Long-Evans Tokushima Fatty (OLETF) rats [35]. The underlying mechanism may be involved in the inhibiting nuclear factor-*κ*B (NF-*κ*B) activation in the myocardium. This biological function directly resulted in downregulation of receptor for advanced glycation end products and connective tissue growth factor (CTGF) expression [35], which have been convinced to play a key role in cardiac fibrosis [50, 51]. As we know, activation of RAAS may also lead to collagen deposition and result in cardiac fibrosis [2, 52]. Research has shown that pioglitazone activation of PPAR*γ* can attenuate cardiac fibrosis in diabetic rats and partly ameliorates cardiac remodeling and function by suppressing activity of RAS [36]. The interesting finding is that rosiglitazone is able to decrease cardiac fibrosis and enhance myocardial vascularization in rat offspring programmed by low protein diet during gestation, which may be implicated in rosiglitazone administration which can decrease angiotensin (Ang) II and endothelin- (ET-) 1 and increase endothelial nitric oxide synthase (eNOS) [37]. Moreover, rosiglitazone reduces atrial interstitial fibrosis and AF promotion in the diabetic rabbits via modulating oxidative stress and inflammation [38]. The selective PPAR*γ*, pioglitazone, could attenuate cardiac fibrosis and collagen concentration by upregulating insulin-like growth factor 1 (IGF-1), phosphorylated Akt, and eNOS in OLETF rats [39]. Furthermore, the PPAR*γ* agonist ciglitazone may alleviate MMP-9 and fibrosis and improve end diastolic diameter in diabetic mice hearts [40]. Unfortunately, a recent study in the same animal model gave a negative conclusion that treatment with rosiglitazone had little cardioprotection and there is no indication for the regulation of NF-*κ*B signaling pathway [41]. But the combination of rosiglitazone and losartan obviously attenuated the interstitial fibrosis and collagen deposition of the heart by inhibiting TGF*β* and tumor necrosis factor-*α* (TNF*α*), along with the proinflammatory cytokines, interleukin- (IL-) 1*β*, and IL-6 [41]. Therefore, the authors declared that the benefit may be not derived from the activation of PPAR*γ*. In addition, combination treatment with rosiglitazone and felodipine could improve the metabolic abnormalities and decrease TNF*α*, TGF*β*, collagen I, and collagen III and increased MMP-2, while treatment with rosiglitazone alone had no effect on attenuating the hypertension and only exerted a minimal effect on reducing cardiac fibrosis and improving dyslipidemia and hyperglycemia in diabetic hypertensive rats [28]. Thus, on one hand, whether the activation of PPAR*γ* which could attenuate myocardial fibrosis remains unclear, the improving of cardiac function may not be related to the attenuation of cardiac fibrosis. On the other hand, the discrepancy results may partly be due to the dosage and length of observation time. Thirdly, the selective ligand, rosiglitazone, presents more discrepancy in the published data, so different structure of selective ligand may show different biological function. More investigations are needed to clarify these perplex.

It has been reported recently that the muscle specific ubiquitin ligase muscle ring finger-2 (MuRF2) and MuRF3 regulate PPAR*γ*1 activity to protect against diabetic cardiomyopathy [53, 54]. Although MuRF2^−/−^ hearts have significant increases in fibrosis and PPAR*γ*1-regulated cardiac genes, the expression of PPAR*γ*1 mRNA has no differences in MuRF2^−/−^ hearts and wild-type mice. Unfortunately, only minimal amount of fibrosis was detected in MuRF3^−/−^ hearts and has no differences compared to wild-type controls. Furthermore, PPAR*γ*1 target genes showed increases in both MuRF3^−/−^ and wild-type hearts, but the mRNA expression levels have no differences between the two groups. Thus it can be seen that MuRF2 and MuRF3 inhibit cardiac PPAR isoforms expression to protect against high fat diet-induced diabetic cardiomyopathy, which mainly improve systolic dysfunction and attenuate left ventricular mass and heart weight but do not include cardiac fibrosis. Therefore, more research needs to prove the role of different PPAR*γ* subtypes in myocardial fibrosis.

### 3.2. Hypertension

There is considerable evidence regarding arterial hypertension which leads to cardiac hypertrophy and myocardial fibrosis [10, 55]. For this reason, it is significant to explore novel strategies to protect the hypertension related cardiac remodeling [56]. Fortunately, despite low expression in the heart, PPAR*γ* acts as a functional antifibrogenic factor in hypertensive heart disease [42]. Recent studies have indicated that treatment with the PPAR*γ* activators resulted in the reduction of ECM deposition and cardiac fibrosis, while PPAR*γ* antagonist GW9662 or T0070907 reversed these changes [10, 42, 43]. In addition, a significant negative correlation was observed between myocardial interstitial fibrosis and mRNA expression of PPAR*γ* [56]. Furthermore, mice with a dominant-negative point mutation in PPAR*γ* (P465L) developed significantly more severe cardiac fibrosis to Ang II-induced hypertension [57].

Despite the fact that the role of PPAR*γ* in chronic pressure overload-induced cardiac fibrosis has been hypothesised previously (details are shown in Table 3), the molecular mechanisms are not fully understood. It has been suggested that activation of PPAR*γ* inhibited both the expressions of TGF*β*1 [10, 42–44, 56] and phosphorylation of Smad2/3 [10] in vivo and cultured neonatal rat cardiomyocytes and cardiac fibroblasts. In addition, the PPAR*γ* agonist pioglitazone significantly decreased cardiac inflammatory response by inhibiting NF-*κ*B and activator protein-1 (AP-1) binding activities, the expression of TNF*α*, and the adhesion of platelet endothelial cell adhesion molecule in stroke-prone spontaneously hypertensive rats (SHRSP) [45]. On the other hand, the downregulation of reactive oxygen species (ROS) mediated by an upregulation PPAR*γ* may play a role in pressure overload-induced cardiac fibrosis [46, 47, 56]. However, Shinzato et al. [44] found that ROS production was not improved in SHRSP treated with pioglitazone. Furthermore, long-term administration of pioglitazone attenuates the development of left ventricular (LV) hypertrophy and fibrosis and inhibited phosphorylation of mTOR and p70S6 kinase in the heart, which are likely attributable to both the activation of AMPK signaling through stimulation of adiponectin secretion and the inhibition of Akt signaling [48].

### 3.3. Myocardial Infarction (MI)

Adverse LV remodeling after MI is characterized by myocyte hypertrophy and interstitial fibrosis of the noninfarcted myocardium [58]. Accumulating evidence suggests that angiotensin II receptor blockers (ARBs) induce the activity of PPAR*γ* which inhibit unfavorable LV remodeling [41, 58–60]. PPAR*γ* protein expression is mainly in cardiac myocytes and fibroblasts in the infarcted area three weeks after MI, suggesting the critical role of PPAR*γ* in cardiac fibrosis [59]. A study conducted by Maejima et al. [58] verified that telmisartan effectively inhibits infaust LV remodeling through a reduction of infiltration of macrophages, activation of MMP2 and MMP9, and expression of TGF*β*1, CTGF, and osteopontin, while expression of PPAR*γ* and activation of tissue inhibitor of metalloproteinase-1 (TIMP-1) were enhanced in the noninfarcted myocardium of rats. And in in vitro experiments, they got the similar results. Pioglitazone, a PPAR*γ* activator, has been proved to reduce TNF*α*, TGF*β*, and monocyte chemoattractant protein-1 and attenuate myocyte hypertrophy and interstitial fibrosis in MI mice [61]. This indicated that an anti-inflammatory effect mediated by PPAR*γ* activation plays a critical role in post-MI LV remodeling in rats. More recently, a multicenter randomized double-blind study demonstrated that Qiliqiangxin, a traditional Chinese medicine, ameliorates unfavorable myocardial remodeling after acute MI including improved cardiac function, decreased apoptosis, and reduced fibrosis by increasing PPAR*γ* levels. However, the expression of well-known signaling pathways including Akt, SAPK/Jun NH_2_-terminal kinase phosphorylation (JNK), and ERK was not altered by Qiliqiangxin treatment [62]. Interestingly, Birnbaum et al. showed that pioglitazone is able to limit myocardial infarct size by activating Akt and upregulating cytosolic phospholipase A2 and cyclooxygenase-2 [63]. These suggest that the underlying mechanism may be varied from different drugs, but PPAR*γ* play a critical role in myocardial fibrosis after MI is indisputable. Besides, TZDs also have neutral [64] or detrimental [65] effects on cardiac remodeling or mortality after MI. Therefore, the exact role of TZDs in myocardial remodeling after MI remains controversial and further studies should be done to elucidate the precise effects and mechanisms.

### 3.4. HF

Although the initial indications for PPAR agonist treatment mainly focus on hyperlipidemia and diabetes, there is a growing body of data which suggest that they maybe improve cardiac function with decreased fibrosis, improved contractility, and endothelial function in animal models of systolic HF [66]. In a rabbit model with nonischemic HF induced by combined aortic regurgitation and aortic stenosis, decreased ejection fraction and unfavorable myocardial remodeling including increased collagen volume fraction were observated. Moreover, the activity and expression of NF-*κ*B subunits p65, RhoA, and Rho GTPase significantly increased. Interestingly, all these changes were reversed and the mRNA and protein expression of PPAR*γ* were significantly increased with simvastatin treatment. Based on these results, the authors declared that simvastatin inhibited RhoA and Rho GTPase signaling to restrain NF-*κ*B activation by the PPAR*γ*-dependent pathway, thus attenuating LV hypertrophy and fibrosis [67]. In addition, pioglitazone treatment reduced the duration of atrial fibrillation (AF) and attenuated atrial structural remodeling including atrial fibrosis via attenuating the expression of TNF*α*, TGF*β*1, and ERK but left unaffected p38 and JNK activation in the rabbit model with congestive heart failure [68]. Therefore, it is conceivable that PPAR*γ* activation suppresses cardiac fibrosis by antagonizing inflammatory and hypertrophic signaling pathways. Likewise, PPAR*γ* acts as a modulator of cardiac fibrosis in human as well. Cardiac remodeling occurring in patients with end-stage heart failure due to ischemic cardiomyopathy is related to PPAR activity, whereby inactivation of PPAR*α* and PPAR*γ* would lead to an increase in the production of ET-1 and the presence of cardiac fibrosis [69]. Nevertheless, rosiglitazone treatment had no significant effects on myocardial fibrosis compared with the vehicle group in MI-induced HF rats [70]. This result should raise questions with regard to these models or the particular species at large. Further studies are needed to test the variety and potential mechanisms.

### 3.5. I/R Injury

Early reperfusion of ischemic myocardium is necessary to salvage myocardial tissue from ultimate death. Nevertheless, reperfusion always results in cardiomyocyte death, microvasculature injury, and cardiac fibrosis, which ultimately cause myocardial remodeling and dysfunction [71, 72]. Recently, research has shown that rosiglitazone alleviated I/R injury by inhibiting inflammatory, improving endothelial function, reducing oxidative stress, and calcium overload [33]. Likewise, rosiglitazone treatment can effectively suppress the inflammatory induced by I/R injury and promote myocardial functional recovery [73] with an inhibition of JNK, AP-1 DNA-binding activity, and NF-*κ*B signaling pathway [33, 73]. These data demonstrated that rosiglitazone limits postischemic injury, suggesting an important function for PPAR*γ* in the heart.

Snail, a zinc finger transcription factor, activation induces lung, liver, and kidneys fibrosis [74–76]. Recently, its role in cardiac fibrosis after I/R injury and the probable underlying mechanisms had been identified. Lee and her colleagues [77] found that I/R injury to mouse hearts significantly increases the expression of Snail. In addition, the author showed that the cell source of Snail induction is endothelial cells. Moreover, Snail overexpression-mediated endothelial-to-mesenchymal transition-like cells markedly stimulated fibroblasts to myofibroblasts transdifferentiation via secretion of CTGF. What is more, they found that PPAR*γ* agonist rosiglitazone, a selective Snail suppressor, remarkably suppressed cardiac fibrosis, improved cardiac function, and reduced Snail and CTGF expression in vivo. Based on this, the authors suggested that Snail might be a potential target molecule in the treatment of cardiac fibrosis.

### 3.6. AF

The relevance of atrial fibrosis and AF is well established and the causal relationship between them is interdependent. Atrial fibrosis expedites the development of AF by causing alterations of electrical properties [78]; on the other hand, AF itself promotes atrial fibrosis [79]. Although the underlying mechanisms are not fully understood, inflammation may promote the persistence of AF and atrial remodeling. A study conducted by Chen et al. [80] suggested that the PPAR*γ* mRNA was significantly decreased in the hypertensive AF patients and PPAR*γ* had a negative correlation with inflammatory cytokines TNF*α*, IL-6, and IL-1. The similar results were observed in elderly patients with AF [81]. In addition, pioglitazone was able to attenuate Ang II-induced electrical and structural remodeling by inhibiting both the TGF*β*1/Smad2/3 and the non-Smad TGF*β*1/tumor necrosis factor receptor associated factor 6/TGF*β*-associated kinase 1 signaling pathways in vitro cellular models [82], which adds further evidence to the benefits of PPAR*γ* agonist for the prevention of AF. Thus, PPAR*γ* is at least partly involved in the pathogenesis of AF by regulation of inflammation through the NF-*κ*B pathway; PPAR*γ* agonist is potential useful in suppressing cardiac fibrosis and preventing AF occurrence.

### 3.7. Other CVD Conditions

It has been demonstrated that myocardial fibrosis is a common pathological change in radiation-induced heart diseases [83]. In Sprague-Dawley rats receiving chest radiation, the protein expression of TIMP-1 and TGF*β*1 was higher than that in rats without radiation in the heart; the PPAR*γ* mRNA and protein expression levels are upregulated in heart injured by radiation as well. However, upregulation of PPAR*γ* failed to inhibit the expression of TIMP-1 and TGF*β*1 [84]. Therefore, it is a possible mechanism that PPAR*γ* itself has protective effect in response to radiation-induced heart injury. Regrettably, the authors did not use PPAR*γ* agonists or inhibitors to further discuss its function in radiation-induced heart diseases. Besides, study on experimental animals demonstrated that tenascin-x, an ECM glycoprotein exclusively expressed in fibroblasts, can inhibit myocardial fibrosis via upregulation of TGF*β*1 and downregulation of PPAR*γ* in alcoholic cardiomyopathy [85]. These data suggested that PPAR*γ* plays a crucial role in inhibiting cardiac fibrosis; further understanding of cardioprotection properties of PPAR*γ* activator came from the study of pioglitazone influence on experimental autoimmune myocarditis. The authors suggested that pioglitazone could alleviate cardiac inflammation and fibrosis by inhibiting macrophage inflammatory protein-1*α* expression and modulating the Th1/Th2 balance [86].

Coincidentally, PPAR*γ* shows a pivotal role in multiple other cardiovascular disease states. Singh et al. [87] demonstrated that rosiglitazone relieves cardiac hypertrophy and myocardial fibrosis in a dose-dependent manner possibly through its antioxidant activity in hyperhomocysteinemia rats. Moreover, simvastatin treatment has beneficial effects on augmentation of the PPAR*γ*, PPAR*α* expression, and reducing cardiac interstitial fibrosis biochemical makers including MMP-9 and cathepsin S in apolipoprotein E-deficient mice fed with a high fat diet [88]. More importantly, irbesartan prevents myocardial hypertrophy and fibrosis via activation of the PPAR*γ* and suppression of the TGF*β*-CTGF-ERK signaling in angiotensin-converting enzyme 2 knockout mice [9]. Finally, activation of PPAR*γ* inhibits isoprenaline- induced myocardial fibrosis and remodeling via the NF-*κ*B and MAPKs-dependent mechanism in rats [89–92].

### 3.8. Cardiac Fibroblasts (CFs) Culture In Vitro

Apart from in vivo experiments, PPAR*γ* have been reported to have a number of cardioprotective properties in vivo. Due to a large number of stresses including growth and vasoactive factors, cytokines, and mechanical stimuli [93], fibroblasts proliferate and differentiate into myofibroblasts, a cell type with an increased secretion capacity of ECM [94]. There is convincing evidence that PPAR*γ* ligands, rosiglitazone, pioglitazone, and 15-deoxy-Δ^12,14^-prostaglandin J2, all inhibit Ang II-induced CFs proliferation and differentiation, collagen synthesis, and ECM production [95–97], which are the critical steps in the pathogenesis of cardiac fibrosis. In addition, rosiglitazone can prevent myocardial fibrosis induced by advanced glycation end products in cultured neonatal rat CFs via inhibiting CFs proliferation, decreasing nitric oxide production, and CTGF expression [98]. Collectively, these data suggest that PPAR*γ* activation has an antifibrotic effect. Despite these findings, the underlying mechanisms for the regulatory effects of PPAR*γ* ligands on cardiac fibrosis are ambiguity and the specific role of PPAR*γ* in this process has not yet been fully elucidated. The molecular mechanisms probably involved NF-*κ*B/TGF*β*/Smad2/3 and JNK signaling pathways [95, 99–101].

## 4. Conclusions and Future Prospects

**Figure 2:**
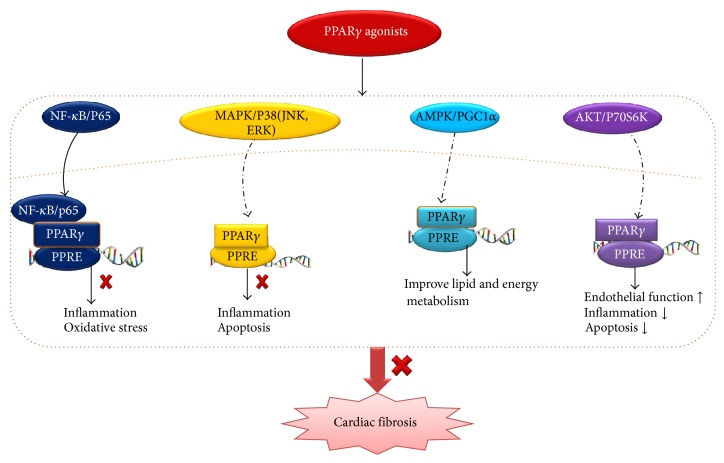
The possible underlying mechanisms involved in PPAR*γ* agonists alleviate cardiac fibrosis. PPAR*γ* agonists show pleiotropy functions associated with inhibiting cardiac fibrosis via variety of signaling pathways. PPAR*γ*: peroxisome proliferator-activated receptor-*γ*; PPREs: peroxisome proliferator response elements; NF-*κ*B: nuclear factor-*κ*B; MAPK: mitogen-activated protein kinase; JNK: Jun NH_2_-terminal kinase phosphorylation; ERK: extracellular signal-regulated kinase; AMPK: adenosine monophosphate-activated protein kinas; PGC1*α*: peroxisome proliferator-activated receptor gamma coactivator-1*α*; AKT: also known as protein kinase B.

Cardiac fibrosis is associated with varied cardiovascular disease and thus is a pivotal determinant of clinical outcome in heart diseases. Although the last decade has seen enormous progress insight into cardiac fibrosis, there is no precise and effective therapy. At the same time, accumulating evidences demonstrate that PPAR*γ* exerts a broad spectrum of biological functions, including the beneficial effects of alleviating myocardial fibrosis. However, the cardioprotection mechanisms are currently not fully established, and the potential mechanisms were shown in Figure 2. Therefore, in-depth understanding of the potential molecular mechanisms of PPAR*γ* and its ligands in preventing cardiac fibrosis may provide valuable information in the design of novel treatment strategies in HF.

Unfortunately, despite many beneficial features of PPAR*γ* agonists, they also exhibit adverse effects associated with long-term use. It has been proposed that PPAR*γ* agonists are not free from side effects including edema, headache, hypoglycemia, myalgia, HF, weight gain, bone fractures, increased risk of MI and mortality, and possibly bladder cancer [13, 14, 17, 102–104]. Rosiglitazone, pioglitazone, and troglitazone have been approved for treatment of type 2 diabetes in clinical practice. Contrary to pioglitazone, rosiglitazone and troglitazone were associated with significant tissue toxicities after a relatively short-term exposure [15, 102]. In addition, the dual PPAR agonist ragaglitazar, MK-0767, naveglitazar, tesaglitazar, and muraglitazar for diabetes have failed due to various safety concerns. Aleglitazar, the most recent dual PPAR*α*/*γ* agonist, has shown a significant dose-dependent reduction in HbA1c and beneficial effects on lipid subfractions [14]. Unfortunately, aleglitazar increased the risks of HF, renal dysfunction, bone fractures, gastrointestinal hemorrhage, and hypoglycemia [105]. Thus, new PPAR*γ*-directed therapeutic modalities should be considered as possible approaches to reducing the adverse events seen with current TZDs. The pan-PRAR agonists bezafibrate, selective PPAR*γ* modulators S26948 and INT131, partial PPAR*γ* agonists balaglitazone, MBX-102, MK-0533, PAR-1622, PAM-1616, KR-62776, and SPPAR*γ* M5, new dual PPAR*α*/*γ* agonists saroglitazar, have a reduced tendency to cause the adverse effects and might be available in clinical management in the near future [14].

PPAR*γ* agonists convey beneficial effects as therapeutic agents for cardiac fibrosis; however, their functions are not fully established yet. As such, PPAR*γ* agonists possess different properties for different species, and the mechanisms by which they attenuate cardiac fibrosis are required in both experimental animal models and humans [106]. Moreover, the adverse side effects of PPAR*γ* agonists and the potential mechanisms responsible for these effects should be clarified in detail, particularly in humans [106]. Last but not the least, it is necessary to focus on interactions between PPAR*γ*-activating agents and other cardiovascular drugs [106]. Intensive research on these targets should be of great assistance to the development of safety and efficacy PPAR*γ* agonists in the near future.

## Figures and Tables

**Table 1:** Tissue and cell distribution of PPAR*γ* mRNA transcripts. Modified from [13].

PPAR*γ* mRNA transcripts	Tissue and cell distribution
PPAR*γ*1	Cardiac muscle, skeletal muscle, kidney, adrenal, spleen, intestine, pancreatic *β*-cells, and vascular smooth muscle cells
PPAR*γ*2	Adipose tissue
PPAR*γ*3	Adipose tissue, colon, and macrophages
PPAR*γ*4	Macrophages
PPAR*γ*5	Macrophages
PPAR*γ*6	Macrophages and adipose tissue
PPAR*γ*7	Macrophages and adipose tissue

**Table 2:** Effects of PPAR*γ* ligands on diabetic related cardiac fibrosis.

Study model	Dose/duration/route	Major cardiac findings and conclusions	Ref.
Male OLETF rats, LETO rats, 20 weeks old	Rosiglitazone 20 mg/kg/d for 20 weeks, gavage	Suppression of RAGE and CTGF expression in the diabetic myocardium appears to contribute to the antifibrotic effect of rosiglitazone	[35]

Male STZ-induced diabetic Sprague-Dawley rats (200 ± 20 g)	Pioglitazone 10 mg/kg/d for 14 weeks, gavage	Activation of the PPAR*γ* signal pathway could repress cardiac fibrosis in diabetic rats and partly improve cardiac remodeling and function by downregulating activity of RAS level	[36]

Male offspring of Wistar rats fed NP diet or LP diet, 3 months old	Rosiglitazone 5 mg/kg/d for three months, gavage	Rosiglitazone showed beneficial effects on rat offspring programmed by low protein diet during gestation decreasing cardiac fibrosis and enhancing myocardial vascularization	[37]

Alloxan-induced diabetic rabbits 1.8–2.5 Kg	Rosiglitazone 2 mg/kg/d for 4 weeks, unclear	Rosiglitazone attenuates arrhythmogenic atrial structural remodeling and atrial fibrillation promotion	[38]

Male OLETF rats, LETO rats, 20 weeks old	Pioglitazone 10 mg/kg/d for 20 weeks, per orem	Activation of PPAR*γ* may decrease collagen concentration and reduce cardiac fibrosis by exerting regulatory effects on cardiac telomere biology	[39]

Male WT, CBS^+/+^, CBS^+/−^, and Ins2^+/−^/CBS^+/−^ rats, 20 weeks old	Ciglitazone 3 mg/kg/d for 4 weeks, orally	Treatment with ciglitazone alleviated MMP-9 activity and fibrosis and improved end diastolic diameter	[40]

Male OLETF rats, LETO rats, 28 weeks old	Rosiglitazone 3 mg/kg/d and losartan 5 mg/kg/d for 12 weeks, gavage	A combination of rosiglitazone and losartan attenuates myocardial fibrosis and dysfunction	[41]

Male diabetic hypertensive rats 179–219 g	Rosiglitazone 3 mg/kg/d or combination of felodipine 5 mg/kg/d for one month, orally	The combined treatment can improve dyslipidemia and decrease TNF*α*, TGF*β*, collagen I, and collagen III, and increased MMP-2 but within a greater effect than treatment with rosiglitazone alone	[28]

OLETF: Otsuka Long-Evans Tokushima Fatty, LETO: Long-Evans Tokushima Otsuka, RAGE: receptor for advanced glycation end products, CTGF: connective tissue growth factor, WT: wild type, CBS^+/−^: cystathionine beta synthase mutant, Ins2^+/−^: insulin 2 mutant, MMP: matrix metalloproteinases, TNF: tumor necrosis factor, TGF: transforming growth factor, NP: normal protein (19% protein), LP: low protein (5% protein), STZ: streptozotocin, and RAS: renin-angiotensin system.

**Table 3:** Effects of PPAR*γ* ligands on hypertension related cardiac fibrosis.

Study model	Dose/duration/route	Major cardiac findings and conclusions	Ref.
Male SHR and WKY rats, 8 weeks old Cell culture: CFs form SD rats, 1-2 days old	Curcumin 100 mg/kg/d or curcumin 100 mg/kg/d plus GW9662 10 mg/kg/d for 12 weeks, gavage	Curcumin attenuates cardiac fibrosis in SHRs and inhibits Ang II- induced production of CTGF, PAI-1, ECM, TGF*β*1, and phosphorylation of Smad2/3 in CFs in vitro	[10]

Male DnTGF*β*RII and WT C57BL/6 mice, 8–10 weeks old subjected to TAC	Rosiglitazone 10 mg/kg/d or T0070907 1.5 mg/kg/d for 3 weeks, gavage	Downregulation of endogenous PPAR*γ* expression by TGF*β* may be involved in pressure overload-induced cardiac fibrosis	[42]

Male Wistar rats, weights 250–300 g subjected to abdominal aortic banding at 4 weeks after ligation Cell culture: CFs form Wistar rats, 1–3 days old	Rosiglitazone 6 g/kg/d or GW9662 0.2 g/kg/d 2 h prior to rosiglitazone 6 g/kg/d for 1 week, intraperitoneal injection	Activation of PPAR*γ* significantly inhibited cardiac remodeling by suppression the expressions of Brq1 and TGF*β*1 through the NF-*κ*B pathway	[43]

Male SHRSP and WKY rats, 24 weeks old	Pioglitazone 10 mg/kg/d for 8 weeks, mixed with food	Pioglitazone decreased interstitial fibrosis and number of myofibroblasts; mRNA levels of collagen I and BNP; MMP2 activity and protein level of CTGF. However, the mRNA level of collagen III and TGF*β*1, MMP9 activity, and ROS production were not improved	[44]

Male SHRSP, 6 weeks old	Pioglitazone 10 mg/kg/d for 20 weeks, mixed with food	Subepicardial interstitial fibrosis, left ventricular NF-*κ*B and AP-1 binding activities, the TNF*α* expression, and the adhesion of PECAM were decreased by pioglitazone treatment	[45]

Male SHRSP and WKY rats, 11 weeks old	Pioglitazone 1 mg/kg/d or 2 mg/kg/d, candesartan 0.3 mg/kg/d for 4 weeks, gavage	Pioglitazone suppressed cardiac inflammation and fibrosis and reduced vascular endothelial dysfunction by inhibition of cardiovascular NADPH oxidase, and the combination of pioglitazone and candesartan exerted more beneficial effects	[46]

Male C57BL/6J rats, 8 weeks old subjected to abdominal aortic banding	Ciglitazone 2 mg/kg/d for 4 weeks, administered in drinking water	Ciglitazone decreased interstitial and perivascular fibrosis and inhibition of an induction of NOX4, iNOS, MMP-2/MMP-13 expression, and collagen synthesis/degradation	[47]

Male inbred Dahl salt- sensitive rats, 7 weeks old	Pioglitazone 2.5 mg/kg/d for 4 weeks, gavage	Pioglitazone treatment ameliorated LV hypertrophy and fibrosis and improved diastolic function by activating AMPK signaling and inhibiting Akt signaling.	[48]

DnTGF*β*RII: dominant-negative mutation of the human TGF*β* type II receptor, WT: wild type, TGF: transforming growth factor, TAC: transverse aortic constriction, CFs: cardiac fibroblasts, NF-*κ*B: nuclear factor-*κ*B, SHR: spontaneously hypertensive rats, WKY: Wistar Kyoto rats, SD: Sprague-Dawley, CTGF: connective tissue growth factor, PAI-1: Plasminogen activator inhibitor-1, ECM: extracellular matrix, SHRSP: stroke-prone spontaneously hypertensive rats, BNP: brain natriuretic peptide, MMP: matrix metalloproteinases, ROS: reactive oxygen species, NADPH: nicotinamide adenine dinucleotide phosphate, NOX4: nicotinamide adenine dinucleotide phosphate oxidase 4, iNOS: inductive nitric oxide synthase, AP-1: activator protein-1, TNF: tumor necrosis factor, PECAM: platelet endothelial cell adhesion molecule, and AMPK: adenosine monophosphate-activated protein kinas.

## Abbreviations

PPAR*γ*: Peroxisome proliferator-activated receptor-*γ*
CVD: Cardiovascular disease
ECM: Extracellular matrix
RAAS: Renin-angiotensin-aldosterone system
MMP: Matrix metalloproteinases
TGF*β*: Transforming growth factor beta
HF: Heart failure
PPREs: Peroxisome proliferator response elements
AF-1: Activation function-1 motif
DBD: DNA-binding domain
LBD: Ligand bind domain
I/R: Ischemia/reperfusion
TZDs: Thiazolidinediones
T2DM: Type 2 diabetes mellitus
OLETF: Otsuka Long-Evans Tokushima Fatty
NF-*κ*B: Nuclear factor-*κ*B
CTGF: Connective tissue growth factor
Ang II: Angiotensin II
ET-1: Endothelin-1
IGF-1: Insulin-like growth factor 1
eNOS: Endothelial nitric oxide synthase
TNF*α*: Tumor necrosis factor-*α*
IL-1*β*: Interleukin-1*β*
MuRF2: Muscle specific ubiquitin ligase muscle ring finger-2
AP-1: Activator protein-1
SHRSP: Stroke-prone spontaneously hypertensive rats
ROS: Reactive oxygen species
LV: Left ventricular
MI: Myocardial infarction
ARB: Angiotensin II receptor blocker
TIMP-1: Tissue inhibitor of metalloproteinase-1
JNK: Jun NH_2_-terminal kinase phosphorylation
AF: Atrial fibrillation
CFs: Cardiac fibroblasts.
